# Meta-Analysis of Brain Gene Expression Data from Mouse Model Studies of Maternal Immune Activation Using Poly(I:C)

**DOI:** 10.3390/genes12091363

**Published:** 2021-08-30

**Authors:** Aodán Laighneach, Lieve Desbonnet, John P. Kelly, Gary Donohoe, Derek W. Morris

**Affiliations:** 1 Centre for Neuroimaging, Cognition and Genomics, Discipline of Biochemistry and School of Psychology, National University of Ireland Galway, H91 TK33 Galway, Ireland; a.laighneach1@nuigalway.ie (A.L.); gary.donohoe@nuigalway.ie (G.D.); 2 Discipline of Pharmacology and Therapeutics, National University of Ireland Galway, H91 TK33 Galway, Ireland; lieve.desbonnet@nuigalway.ie (L.D.); john.kelly@nuigalway.ie (J.P.K.)

**Keywords:** maternal immune activation, mouse model, gene expression, schizophrenia, autism spectrum disorder, cognition

## Abstract

Maternal immune activation (MIA) is a known risk factor for schizophrenia (SCZ) and autism spectrum disorder (ASD) and is often modelled in animal studies in order to study the effect of prenatal infection on brain function including behaviour and gene expression. Although the effect of MIA on gene expression are highly heterogeneous, combining data from multiple gene expression studies in a robust method may shed light on the true underlying biological effects caused by MIA and this could inform studies of SCZ and ASD. This study combined four RNA-seq and microarray datasets in an overlap analysis and ranked meta-analysis in order to investigate genes, pathways and cell types dysregulated in the MIA mouse models. Genes linked to SCZ and ASD and crucial in neurodevelopmental processes including neural tube folding, regulation of cellular stress and neuronal/glial cell differentiation were among the most consistently dysregulated in these ranked analyses. Gene ontologies including K+ ion channel function, neuron and glial cell differentiation, synaptic structure, axonal outgrowth, cilia function and lipid metabolism were also strongly implicated. Single-cell analysis identified excitatory and inhibitory cell types in the cortex, hippocampus and striatum that may be affected by MIA and are also enriched for genes associated with SCZ, ASD and cognitive phenotypes. This points to the cellular location of molecular mechanisms that may be consistent between the MIA model and neurodevelopmental disease, improving our understanding of its utility to study prenatal infection as an environmental stressor.

## 1. Introduction

Prenatal infection is among the most well-studied environmental risk factors for abnormal neurodevelopment [1], presenting a significant risk to public health. Maternal immune activation (MIA) in mouse using Poly (I:C) is a well-established model of human prenatal infection. The offspring of dams that experience immune activation during pregnancy show a comparable cognitive and social behaviour profile to schizophrenia (SCZ) and other neurodevelopmental disorders such as autism spectrum disorder (ASD) including impaired social interaction [2,3,4,5,6], increased fear response [4], stereotyped behaviour [5] and decreased performance in cognitive tests including working memory [3,6,7,8] and probabilistic learning [2].

Many studies of MIA models include analysis of gene expression to investigate the effect of infection on molecular genetic function within the brains of offspring. Technologies for transcriptome-wide analysis including RNA sequencing (RNA-seq) and microarrays facilitate the detection of gene expression changes between experimental treatment groups and controls. These techniques provide insights into the effect of MIA on brain gene expression. Individual studies find MIA to elicit several changes to the expression of genes involved in ion channel signalling (notably K^+^ and Ca_2_^+^) [2,9], myelin functionality and stability [3,10], neurotransmission (notably glutamatergic and GABAergic) [2,4], synapse structure and function [9] and nucleic acid binding and maintenance [5].

Investigating expression changes for individual genes alone can lack context. Most biological processes are influenced by multiple genes. For this reason, Gene Ontology (GO) analysis can be usefully performed on sets of differentially expressed genes (DEGs) in order to find biological pathways, cellular compartments and molecular functions that may be disrupted as a result of MIA. In combination with specific genetic and ontological changes, investigating vulnerable cell types can also crucially inform on the biological impact of gene expression changes. Large single cell transcriptomics studies employing single cell RNA-seq (scRNA-seq) [11,12] now provide the essential framework to determine what cell types are enrichment for DEGs reported in MIA studies. Reviewing the molecular function of enriched cell types can provide cell and tissue-level context for the effect of MIA. Lastly, it is interesting to relate the gene expression changes observed in MIA models to what we know about the genetic basis of neurodevelopmental disorders from human studies. These include genome-wide association studies (GWAS) of common genetic variants and sequencing studies of rare and de novo mutations, which together identify the risk genes for these disorders.

Our aim was to combine and meta-analyse gene expression data from multiple studies of MIA models to identify genes that showed consistent changes in expression across those studies. Based on these data, we next identified biological processes and individual cell types associated with these genes. We further investigated if these genes, processes and cell types were enriched for risk genes reported in human studies of neurodevelopmental phenotypes. Our rationale for doing so was that significant overlap between human studies and the MIA model could point to cell types and molecular mechanisms that are vulnerable to genetic variation and environmental stress, and may underpin the cognitive and social changes common to both.

## 2. Materials and Methods

### 2.1. Data Selection

A comprehensive literature search using NCBI PubMed (https://pubmed.ncbi.nlm.nih.gov (accessed on 7 January 2021)) identified 33 original research papers reporting on MIA models up until 31 December 2020. Of these, twelve used Poly(I:C) as the stimulating agent in mouse and the number was further reduced to nine when only considering those studies that included transcriptome-wide analysis of gene expression using either RNA-seq or microarray. Three studies were excluded based on their experimental parameters; analysis of foetal brain tissue rather than adult brain [13], analysis non-neuronal cells [6] and a model using a pharmacological rescue [14]. Data were not readily available online or through author correspondence for a further three studies [5,7,15]. That left three C57BL6 mouse model Poly(I:C) studies with gene expression data that contained four usable datasets. These were RNA-seq data from frontal cortex (FC) [2], RNA-seq data from amygdala (AM) [4] and microarray data from medial pre-frontal cortex (mPFC) and from nucleus accumbens (NAc) [3]. To remain consistent with the other datasets, only the RNA-seq data from the first generation offspring (F1) were used for analysis of AM.

### 2.2. Analysis of Gene Expression Data

RNA-seq raw data were obtained in FASTQ format. Trimmomatic v0.33 [16] was used to remove low-quality and adapter sequences from the single-end reads (LEADING:3, TRAILING:3, SLIDINGWINDOW:4:15, MINLEN:36). Salmon v0.8.2 [17] was used to quasi-map and quantify reads. The DEseq2 v1.24.0 [18] R package was used to test genes for differential expression. Microarray data were obtained in CEL format. Pre-processing of CEL files was performed using oligo v1.48.0 [19] R package. The limma v3.40.6 [20] R package was used to test genes for differential expression at a false discovery rate (FDR) < 0.1. Genes were categorised as DEGs for Poly(I:C) treatment compared to control at an FDR < 0.1. No expression fold change constraint was applied. 

### 2.3. Gene Ontology

Sets of DEGs were subjected to Gene Ontology (GO) statistical overrepresentation tests using ConsensusPathDB (http://cpdb.molgen.mpg.de/ (accessed on 20 January 2021)) [21]. Overrepresented biological processes, molecular functions and cellular compartments were considered significant at an FDR-corrected *p*-value (*q*-value) of < 0.05. Where necessary, the biomaRt 2.40.5 [22] R package was used to convert mouse DEGs to their human orthologues. 

### 2.4. Meta-Analysis Using Gene Ranking

To qualify for the ranking analysis, each unique gene had to be differentially expressed in the same direction in all four datasets (either consistently upregulated or downregulated in Poly(I:C)-treated animals compared to controls, regardless of magnitude). Once genes were separated into upregulated and downregulated lists, they were ordered individually by significance and each gene was given a ranking (the most significant gene was ranked first). The rankings for each were then arithmetically summed or multiplied to produce sum rank and product rank, respectively. The top 20 genes in each of the 4 lists were subjected to a literature search for links to SCZ, ASD and/or neurodevelopment. The top 5% of each list were subjected to GO analysis.

### 2.5. Enrichment of Gene-Sets for Common Genetic Variants Associated with Neurodevelopmental Phenotypes

GWAS summary stats for SCZ (40,675 cases and 64,643 controls) [23], ASD (18,381 cases and 27,969 controls) [24], and the cognitive phenotypes of human intelligence (IQ; 269,867 individuals) [25] and educational attainment (EA; 766,345 individuals) [26] phenotypes were obtained. MAGMA v1.06 [27] was used for gene-set enrichment analysis of sets of DEGs from MIA studies. First, MAGMA annotated the SNP data to genes using the build 37 gene locations (https://ctg.cncr.nl/software/MAGMA/aux_files/NCBI37.3.zip (accessed on 27 January 2021)) and 1000 Genomes European Panel reference (https://ctg.cncr.nl/software/MAGMA/ref_data/g1000_eur.zip (accessed on 27 January 2021)) files, the latter which MAGMA uses to account for linkage disequilibrium (LD) between SNPs. Second, MAGMA generated *p*-values for individual gene reflecting their level of association with a phenotype. Thirdly, using each gene’s association result, MAGMA tested each gene-set for enrichment of genes associated with SCZ, ASD, IQ or EA.

### 2.6. Enrichment of Gene-Sets for De Novo Mutations

Gene-sets were tested for enrichment of de novo mutations found in published exome sequencing studies of SCZ (3394 trios) [28,29,30,31] and ASD (6430 trios) [32] using the denovolyzeR R package [33]. Mutations were classed as synonymous, missense or loss-of-function. Results were then subjected to a competitive test against background de novo mutation rate using a two-sample Poisson rate ratio test. Results were subjected to a Bonferroni multiple testing correction.

### 2.7. Cell Type Enrichment

Data from two separate scRNA-seq studies on the mouse brain (565 cell types [11]) and nervous system (265 cell types [12]) were used to test if different cell types were enriched for DEGs from the MIA studies. Analysis was performed on each gene-set using the expression-weighted cell type enrichment (EWCE) R package [34], which investigated whether the cell types were significantly enriched for a gene-set when weighted by gene expression. Cell types were considered significant after Bonferroni correcting for the number of cell types tested. For comparison to human studies of genetic variation, Functional Mapping and Annotation of Genome-Wide Association Studies (FUMA) cell type enrichment [35] was performed on output files (.raw) of each phenotype from previous MAGMA [27] analysis of GWAS data using the same single cell data. Cell types with *p*-values less than Bonferroni-adjusted significance thresholds for the human data were then compared to those cell types from the MIA analysis.

## 3. Results

Table 1 summarises the main properties of the four datasets used in this analysis. No two datasets have conditions that directly replicate each other. The major differences between the four datasets are due to these studies using four different brain regions, two different techniques for measuring RNA expression, two different Poly(I:C) doses and three different timepoints during gestation for administration of Poly(I:C). The number of DEGs between treatment and control groups at FDR < 0.1 based on our analysis of each dataset varied considerably. Two orders of magnitude separated the number of DEGs found in the FC study (*n* = 39 in total) compared to the mPFC study (*n* = 1628 in total) data. The one consistent factor across the four datasets was that more DEGs were significantly upregulated than downregulated in treatment versus control groups.

### 3.1. Differential Gene Expression

**Figure 1:**
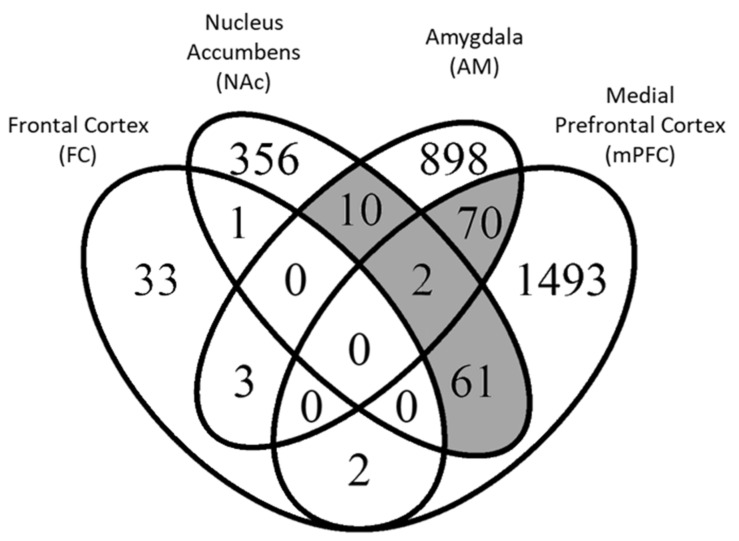
Differentially expressed genes (DEGs) detected at FDR < 0.1 in each respective analysis. Overlapping sets of genes with ≥10 genes (shaded grey) were subjected to overlap gene ontology analysis. The two genes common to Nac, AM and mPFC were *Crabp1* and *Drd3*.

Figure 1 displays the overlap between DEGs across the four datasets. Individual gene results for each dataset are detailed in Supplementary Table S1. There is relatively little overlap between the datasets with no genes differentially expressed in all four datasets and just two genes that were common to three datasets. These were *cellular retinoic acid binding protein 1* (*Crabp1*) and *dopamine receptor D3* (*Drd3*) and only *Crabp1*’s change in expression was in the same direction for all three studies where it was downregulated in treated animals compared to controls. The D3 dopamine receptor plays a role in clinical antipsychotic response [36]. Crabp1 encodes a protein that binds retinoic acid, which is involved in the differentiation of neurons [37,38,39]. Of the 149 genes common to two or more datasets, most were shared between AM and mPFC (*n* = 72), NA and mPFC (*n* = 63), and AM and NAc (*n* = 12). 

**Figure 2:**
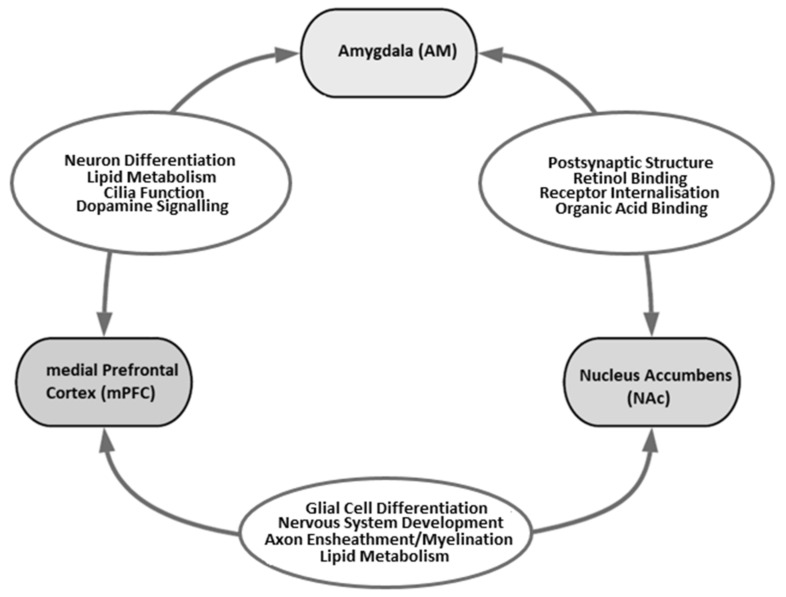
Overlapping DEGs between studies were subjected to GO analysis. GO terms were considered significant at FDR < 0.05. The ovals between two brain regions highlight the GO terms relevant to neurodevelopment that were enriched for DEGs found in both of those brain regions.

These three sets of DEGs that overlapped between studies were subjected to GO analysis. Several GO terms relevant to neurodevelopment were found to be enriched for overlapping DEGs: neuron differentiation, nervous system development, glial cell differentiation, ensheathment of neurons, myelination, synaptic structure and function, and dopamine receptor signalling. Figure 2 illustrates where some of these GO terms emerged in this analysis. Full summaries of GO analysis results can be found in Supplementary Table S2.

### 3.2. Meta-Analysis by Gene Ranking

Separate meta-analyses of upregulated (where genes had higher expression levels in treated animals compared to controls) and downregulated genes were performed next. Nine hundred and forty-seven genes were consistently upregulated, and 428 genes were consistently downregulated in all four datasets. After ranking within each dataset, ranks were then combined across the datasets by two methods, a product of the ranks and a sum of the ranks (from here on termed product rank and sum rank). There was a high degree of consistency between the product rank and the sum rank for the downregulated genes where eleven genes were in the top twenty positions for both methods (Table 2). There was less consistency for the upregulated genes where just four genes were in the top twenty positions for both methods (Table 2). The full meta-analysis results can be found in Supplementary Table S3.

To see what biological processes were enriched for the highest-ranking genes, GO analysis was performed on the top 5% by rank of these upregulated and downregulated genes. Enriched GO terms for upregulated genes in the top 5% for sum rank included K^+^ channel structure and function, synaptic structure, neuron projection and vesicle transport. The full list of significantly enriched GO terms for these gene-sets are in Supplementary Table S4. From Table 2, above, we see that several the highest-ranking genes are driving these enrichments. *Cck*, *Slc4a7,* and *Kcnn1* (positions 1, 4 and 17 of 947 upregulated genes) are members of the enriched neuron projection (GO:0043005) ontology. Furthermore, *Cck* and *Slc17a5* (positions 1 and 15) are members of the enriched presynapse (GO:0098793) ontology. As discussed below, many of the individual highest-ranking genes also have ties to SCZ/ASD including *Cck* (position 1 in summed rank) and Szt2, *Fgf10, Serinc2 and Crhbp* (positions 1, 2, 5 and 6 in product rank), as well as many having ties to crucial neurodevelopmental processes.

### 3.3. Cell-Type Enrichment Analysis

Nine gene-sets were included in this analysis; the four sets of DEGs from the individual studies (as these genes were not subjected to single-cell analysis in the original studies), the three sets of DEGs that overlapped between AM and mPFC, NAc and mPFC, and AM and NAc, and lastly the two set of genes that were consistently upregulated and consistently downregulated across all four studies from the meta-analysis. Each gene-set was analysed in two independent datasets of scRNA-seq gene expression data from the mouse brain [11] (referred to as the Saunders data) and nervous system [12] (referred to as the Zeisel data) to determine which cell types were enriched for genes that are differentially expressed in MIA mouse models. 

There were consistent results between the analyses of the independent Zeisel and Saunders datasets (Table 3). For the individual studies, DEGs from both mPFC and NAc were enriched in oligodendrocytes. DEGs from AM were enriched in inhibitory D1 and D2 medium spiny neurons (MSNs) and the equivalent inhibitory direct/indirect spiny projection neurons (dSPNs/iSPNs; both striatum), and in ependymal cells and choroid plexus cells. For the DEGs that overlapped between brain regions, the enriched cell types again included oligodendrocytes and D2 MSNs and dSPNs/iSPNs (both striatum). Lastly, following meta-analysis, DEGs that were consistently upregulated across studies were enriched in both excitatory neurons (pyramidal cells) in the cortex and inhibitory interneurons in the hippocampus. Full lists of significantly enriched cell types are provided in Supplementary Tables S5 and S6.

For comparison, we performed analysis using the GWAS results for SCZ, ASD, EA and IQ to identify cell types that are enriched for genes associated with these phenotypes (Supplementary Table S7). Of the cell types enriched from DEGs from the MIA models, several are also enriched for associated genes from these human studies (Table 4). From the individual studies, for mPFC, these were excitatory neurons that are enriched for genes associated with SCZ, ASD and EA, and for AM, these were D1 and D2 MSNs and dSPNs/iSPNs, which are enriched for genes associated with SCZ, EA and IQ. Of the cell types enriched for MIA meta-analysis upregulated genes, several excitatory neurons from the cortex (e.g., pyramidal cells) and hippocampus (e.g., CA1 principal cells) were also enriched for genes associated with SCZ and IQ (Table 4).

### 3.4. Enrichment for Genes Involved in Human Phenotypes

To further investigate the overlap between the gene expression results from the MIA models and results of human GWAS, the same nine gene-sets were directly tested for enrichment for genes containing common genetic variants associated with SCZ, ASD, IQ and EA. Although a small number of nominally significant results were produced, no gene-set enrichment survived correction for multiple testing (Supplementary Table S8). We also tested the nine gene-sets for enrichment for rare de novo mutations found in patients with SCZ and ASD. No gene-sets were found to be significantly enriched for synonymous (syn), missense (mis) or loss-of-function (lof) mutations after the results of competitive tests were corrected for multiple testing (Supplementary Table S9).

## 4. Discussion

Although each study reports findings with respect to its individual conditions, it is also important to consider studies together in order to gain an insight into the reliable changes in gene expression that consistently result from MIA. Identifying consistent gene expression changes caused by MIA such as DEGs in multiple studies, including where genes are up- or down-regulated in the same direction across multiple studies, can provide valuable information about robust genetic alterations in these models. Our first finding of note was that when the literature was fully reviewed and data access explored, there was only a small proportion of published MIA mouse model studies that had fully available transcriptome-wide gene expression data. Only two of the four datasets available were from the same brain region (frontal cortex). Despite this, as gene expression patterns are very often strongly correlated across brain regions, we still proceeded to initially identify genes that had consistent changes in expression across those studies. Although analysis of the FC data shows an apparently diminished biological response compared to the other datasets, the original study found behavioural effects consistent with ASD as well as transcriptomic changes involving glutamatergic neurotransmission, mTOR signalling and K^+^ ion channel activity, indicating a true relevant neurobiological response. No genes were differentially expressed in all four datasets, likely due to the small number of DEGs in the FC dataset, consistent with the original study [2]. Among the other three datasets, one gene, *Crabp1*, was differentially expressed in the same direction, with reduced expression observed following MIA.

When the datasets were meta-analysed, five upregulated genes (*Fgf10*, *Cck*, *Crhbp*, *Igfbp7*, *Napepld*) and one downregulated gene (*Zeb1*) among the top 20 genes were found to have links to SCZ. Higher concentration of *Fgf10* product is associated with increased positive and negative syndrome scale (PANSS) total score in humans [40]. The *Cck* gene has a neuropeptide product in which the human orthologue is found to have abnormal levels of mRNA in the entorhinal cortex of schizophrenia patients [41]. A receptor gene for human *CCK* gene product (CCK-AR) may also be associated with positive symptoms of schizophrenia, particularly auditory hallucinations [42,43,44,45]. *CRHBP* is a gene involved in the HPA-axis found to have links with suicide attempt [46] and alcohol use disorder [47] comorbidities in schizophrenia. Serum levels of the *IGFBP7* gene product was found to be significantly lower in patients with schizophrenia compared to controls [48]. *NAPEPLD* contains a polymorphism associated with SCZ in a Han Chinese sample [49]. Finally, *ZEB1* was implicated in human schizophrenia in a GWAS region-based analysis that analysed signals from independent 100 kb regions [50].

Three downregulated genes (*Adcy5*, *Dock10*, *Gli3*) and three upregulated genes (*Szt2*, *Maf*, *Serinc2*) among the top 20 genes have possible links to ASD. Of the downregulated genes, each has a link to ASD or autism-like behaviour through deletion. Loss of *Adcy5* in mouse dorsal striatum lead to autism-like behaviour [51]. A rare deletion in human *DOCK10* following an autosomal recessive inheritance pattern was attributed as being causal of ASD [52]. Lastly, a human deletion in *GLI3* was associated with causing subclinical autism in the father of a child with ASD and Greig cephalopolysyndactyly syndrome [53]. Of the upregulated genes, a *SZT2* mutation was reported to be causal of a syndrome incorporating autistic features as well as intellectual disability, epilepsy and developmental delay [54], *Serinc2* is a proposed ASD candidate following a chromosomal array [55] and *Maf* has variants associated with Aymé-Gripp syndrome, which includes ASD as a characteristic [56]. 

Many of the top genes have associations with relevant disorders and important neurodevelopmental processes. The *Szt2* gene appeared to be strongly implicated in seizure threshold in mouse [57] and mutations in its human orthologue *(SZT2*) may be involved in macrocephaly, developmental delay, pharmacoresistant epilepsy and intellectual disability phenotypes [54,58,59,60,61,62,63]. Variants in the human orthologue of *Serinc2* [64,65] and *Kcnn1* [66] are strongly implicated in alcohol dependence. Many of the genes are involved in neurodevelopmental or neuroprotective processes. The *Rybp*, *Scube3* and *Ifrd1* (also known as *PC4*) gene products are found in high levels in the developing neural tube, with *Rybp* playing a crucial role in mouse neural tube closure in early development [67,68,69]. Genes including *Rybp*, Slc4a7, *Fgf10*, *Zeb1*, *Hmgn2* and *Gli3* play a role in neuronal differentiation [70,71,72,73,74,75]. The *Fgf10* gene also plays a role in axonal regeneration and recovery [76] as well as being crucial in the mediation of dendrite outgrowth of glutamatergic neurons in mouse [77]. Another two top genes share similar roles; *Rem2* has a function in forming glutamate synapses and dendrite outgrowth [78], while *Rgmb* plays a role in axonal regeneration [79]. Some of the top genes are also involved in facilitating neuroprotection from excitotoxic or oxidative stress. *Fgf10* provides protection against ischemia-driven oxidative stress [76], while *Syt10* and *Slc4a7* act to protect neurons from excitotoxic injury [80,81]. 

Gene ontology analysis of the highest-ranking genes revealed enrichments for genes involved in ontologies of synaptic structure, neuron projection and vesicle transport, all of which are crucial to the proper function and maintenance of synapses. Synapse function is implicated in schizophrenia in studies based on both post-mortem analysis of human brains [82] and genetic vulnerability based on common variants [83]. Similar findings are valid for ASD, with studies also supporting postmortem [84] and genetic [85] synapse vulnerability. Genes found to be both consistently overexpressed and involved in enriched ontologies including *Cck*, *Slc4a7*, *Slc17a5*, *Napepld* and *Kcnn1* may be interfering with the ability to maintain normal synaptic structure and function and leading to aberrant neurotransmission and ultimately, behaviour.

Using scRNA-seq data to identify the cell types that are enriched for DEGs was not performed in any of the original studies. This analysis provided some interesting insights. Firstly, several the DEGs that are driving the cell type enrichments are also driving the GO enrichments, indicating cell types where different biological process may be disrupted. For example for the mPFC^+^ NAc DEGs, all seven genes *(Serinc5*, *Ndrg1*, *Myrf*, *Lpar1*, *Pllp*, *Mal*, *Bcas1*) that are members of the enriched “ensheathment of neurons” GO term and four out fo the five gene members (*Erbb3*, *Myrf*, *Lpar1*, *Ndrg1*) of the enriched “glial cell differentiation” GO term are highly expressed in the enriched cortical and hippocampal oligodendrocytes from the Saunders data. All of these genes, with the exception of Mal, are also among the top 5% of genes specifically expressed in the enriched MOL2 oligodendrocyte cell type in the Zeisel data. Three of the seven genes from the mPFC^+^ AM overlap (*Ccdc39*, *Dnah3*, *Dnali*) that are members of the “cilium” GO term are in the top 5% of genes specifically expressed in the enriched EPEN ependymal cell type in the Zeisel data. Finally, one of two DEGs from the mPFC^+^ AM overlap (*Adcy5*) is a member of the enriched “adenylate cyclase-activating dopamine receptor signalling pathway” GO term and this gene is highly expressed in dSPN and iSPN neurons among dopaminergic striatal cell types in the Saunders data, while *Adcy5* is also in the top 1% of genes specifically expressed in both of the enriched dopaminergic striatal MSN cell types (MSN2, MSN3) from the Zeisel data. Although the enriched lipid metabolism GO terms that could be linked to a cell type, a disruption in lipid metabolism may have a link to human SCZ development. Recent evidence showed baseline serum lipid differences between individuals at clinical high risk for psychosis (CHR) and healthy control, as well as distinct serum lipid profiles between CHR individuals who went on to develop psychosis and those who did not after 5-year follow up [86].

Several of the cell types found to be enriched for DEGs from MIA studies are also enriched for genes associated with SCZ, ASD or cognitive phenotypes in human studies. These include excitatory glutamatergic neurons (pyramidal cells) in the cortex and CA1 principal cells, hippocampal interneurons and cells of the entorhinal cortex. Studies of SCZ implicate the hippocampus at the anatomical level, with meta-analysis finding reductions in hippocampal volume in both first-episode psychosis and chronic schizophrenia compared to healthy control [87]. The entorhinal cortex is a region in which there is strong imaging-based evidence of anatomical difference in SCZ [88]. Hippocampal interneurons also show apparent compositional and transcriptional abnormalities in SCZ, with decreases in crucial marker mRNAs including somatostatin, parvalbumin and glutamic acid decarboxylase (GAD) without overall reductions in cell numbers found [89]. Glutamatergic cells from the medulla (Nucleus of the Solitary Tract (NTS)) were enriched for mPFC DEGs and for genes associated with SCZ, ASD and EA. Although little evidence directly links the NTS to SCZ, a possible therapeutic link exists. Transcutaneous stimulation of the vagus nerve, which projects to the NST, has been proposed as an alternative SCZ therapy [90], given some efficacy as a rescue in an animal model of SCZ [91], but not currently reproduced in human trials [92]. Lastly, inhibitory GABAergic cells from the striatum (MSNs/dSPNs/iSPNs) were enriched for AM DEGs and for genes associated with SCZ, EA and IQ. These cells are involved in dopamine-signalling tracts often thought to be disrupted in SCZ [93]. 

Despite these cell type links between the MIA transcriptomic data and human GWAS studies, there was no direct overlap between MIA DEGs and genes associated with SCZ, ASD, IQ and EA. Furthermore, no enrichments for rare de novo mutations contributing to SCZ or ASD were found among MIA DEGs. This result in the context of the cell type enrichment similarities suggests that the additional risk for SCZ caused by maternal infection may be mediated through a smaller set of genes regulating a small number of vulnerable cell types. Larger genome-wide investigations may not be able to effectively detect this signal in isolation. It is possible that other risk factors may induce similar cell-specific enrichments that, when integrated with MIA, would facilitate the detection of a more generalised risk spread across a larger set of genes. This suggests that while the same cell types could be affected by genetic variation in the human disorders and environmental stress in the MIA models, underpinning the cognitive and social behaviours common to both, it is not the same genes that are affected.

There are several limitations to the approach used in this study. First, the low number of datasets make it difficult to generalise findings to future studies of MIA. Second, two datasets are from the same study, performed under identical conditions, leading to possible biases in the data. Third, although the need for all genes to be expressed in the same direction is a robust measure, it suffers from a general lack of power and misses out on many genes that may have been differentially expressed in the same direction in at least three studies. Fourth, expression fold changes were not taken into consideration when finding and ranking unidirectional genes. Finally, gene expression data are derived solely from male mice, and the confounding effects of sex on the outcome cannot be studied.

## 5. Conclusions

In summary, this study combined four RNA-seq and microarray datasets in an overlap analysis and ranked meta-analysis in order to investigate genes, pathways and cell types dysregulated in MIA mouse models. Genes linked to SCZ and ASD and crucial in neurodevelopmental processes including neural tube folding, regulation of cellular stress and neuronal/glial cell differentiation were among the most consistently dysregulated in these ranked analyses. Gene ontologies including K+ ion channel function, neuron and glial cell differentiation, synaptic structure, axonal outgrowth, cilia function and lipid metabolism were enriched for DEGs. Single-cell analysis identified excitatory and inhibitory cell types in the cortex, hippocampus and striatum that may be affected by MIA and are also enriched for genes associated with SCZ, ASD and cognitive phenotypes, pointing to the location of molecular mechanisms that may be consistent between the MIA model and the neurodevelopmental diseases that the model is used to study. Understanding the link between genetics, environment, and this early neurodevelopment, which is facilitated by molecular analysis of MIA models, is essential for study of neurodevelopmental disorders.

## Figures and Tables

**Table 1:** Summary parameters of individual datasets.

Study Reference	Technique	Brain Region	# Controls/Treatments	Poly(I:C) Dose; Day of Admin. *	# Up-Regulated DEGS (FDR < 0.1)	# Down-Regulated DEGs (FDR < 0.1)
[2]	RNA-seq	Frontal Cortex (FC)	8/9	20 mg/kg; E12.5	21	17
[3]	Microarray	Prefrontal Cortex (mPFC)	6/6	5 mg/kg; GD17	1042	586
[3]	Microarray	Nucleus Accumbens (Nac)	6/6	5 mg/kg; GD17	225	206
[4]	RNA-seq	Amygdala (AM)	6/5	5 mg/kg; GD9	521	462

* E = embryonic day, GD = gestational day, # = Number of.

**Table 2:** Top 20 positioned genes in sum and product rank.

Position	Product Rank (Down)	Product Sum (Down)	Product Rank (Up)	Product Sum (Up)
1	Rybp	Scube3	**Szt2**	**Cck**
2	Tdrp	Hs6st2	**Fgf10**	Mical2
3	Dach1	Dach1	Ttc13	Mroh1
4	Scube3	Syt10	Slc9b2	Slc4a7
5	Hs6st2	Rybp	**Serinc2**	Ifrd1
6	Itga5	Hmgn2	**Crhbp**	Dysf
7	Tceal5	Ankef1	Rgmb	Ociad2
8	Syt10	Th	**Maf**	Fam126b
9	**Adcy5**	Ogfr	Ints10	Elmo2
10	**Dock10**	Mid1	**Igfbp7**	Hapln4
11	Hmgn2	**Dock10**	Letmd1	Anxa11
12	**Zeb1**	B230118H07Rik	**Napepld**	Rnaseh2a
13	Ass1	**Gli3**	Aifm3	Arl5a
14	Myl12b	Aim2	Tmem25	Serpinb8
15	**Gli3**	Ass1	Rnaseh2a	Slc17a5
16	Serinc5	Ecscr	Mospd1	Siah3
17	BC005624	Bex4	Cblb	Kcnn1
18	Bex4	Pbx3	Tnpo1	Zfp697
19	Rem2	Itga5	Mroh1	**Maf**
20	Smndc1	Chmp6	Ifrd1	Mettl1

Note: Genes with link to schizophrenia (SCZ) or autism spectrum disorder (ASD) are highlighted in bold. Genes with links to neurodevelopmental process are underlined.

**Table 3:** Significantly enriched cell types as determined by expression-weighted cell type enrichment (ewce) using single cell gene expression data.

Gene-Set		Enriched Cell Types in Zeisel Data (*n* = 265 Cell Types)	Enriched Cell Types in Saunders Data (*n* = 565 Cell Types)
Individual Study DEGs	mPFC	**-Oligodendrocytes** -Excitatory Neurons (Hindbrain) -Inhibitory D2 Medium Spiny Neurons (Striatum)	**-Oligodendrocytes**
	NAc	**-Oligodendrocytes**	**-Oligodendrocytes**
	AM	**-Inhibitory D1 & D2 Medium Spiny Neurons (Striatum)** **-Ependymal Cells** **-Choroid Plexus Cells** -Hypendymal Cells -Vascular Leptomeningeal Cells	**-Inhibitory direct/indirect Spiny Projection Neurons (Striatum)** **-Ependymal Cells** **-Choroid Plexus Cells** -Endothelial Cells
Overlap DEGs	mPFC + NAc	**-Oligodendrocytes**	**-Oligodendrocytes**
	mPFC + AM	**-Inhibitory D2 Medium Spiny Neurons (Striatum)** -Ependymal Cells	**-Inhibitory direct/indirect Spiny Projection Neurons (Striatum)**
Meta-analysis DEGs	Up-regulated	**-Excitatory Neurons, Pyramidal Cells (Cerebral Cortex)** **-Inhibitory Interneurons (Hippocampus)** -Inhibitory Interneurons (Hypothalamus)	**-Excitatory Neurons, Deep-layer Pyramidal cells (Frontal Cortex)** -Excitatory Neurons (Posterior Cortex) -Excitatory Neurons, CA1 Principal Cells (Hippocampus) -Excitatory Neurons, Entorhinal Cortex Cells (Hippocampus) **-Inhibitory Interneurons (Hippocampus)**
	Down-regulated	-Microglia	-Endothelial Stalk Cells

Enriched cell types highlighted in **bold** are consistent between the two analyses of independent single cell datasets. DEGs = Differentially expressed Genes, mPFC = medial Prefrontal Cortex, NAc = Nucleus Accumbens, AM = Amygdala.

**Table 4:** Cell types that are enriched for both DEGs from MIA models and genes associated with schizophrenia (SCZ), autism spectrum disorder (ASD), intelligence (IQ) or educational attainment (EA) from genome-wide association studies (GWAS).

Gene-Set		Significant Cell Types from Zeisel That Are Common between Mouse MIA and Human GWAS	Significant Cell Types from Saunders That Are Common between Mouse MIA and Human GWAS
Individual Study DEGs	mPFC	-Excitatory Neurons (Hindbrain) **[SCZ, ASD, EA]**	No cell types
	AM	-Inhibitory D1 & D2 Medium Spiny Neurons (Striatum) **[EA]**	-Inhibitory direct/indirect Spiny Projection Neurons (Striatum) **[SCZ, EA, IQ]**
Meta-analysisDEGs	Upregulated	-Excitatory Neurons, Pyramidal Cells (Cerebral Cortex) **[SCZ, IQ]**	-Excitatory Neurons, Deep-layer Pyramidal cells (Frontal Cortex) **[SCZ, IQ]** -Excitatory Neurons (Posterior Cortex) **[SCZ, IQ]** -Excitatory Neurons, Entorhinal Cortex Cells (Hippocampus) **[SCZ, IQ]** -Excitatory Neurons, CA1 Principal Cells (Hippocampus) **[IQ]**

## Data Availability

Data were directly downloaded from published studies and all additional generated data is contained within manuscript and supplementary data of this study.

## Supplementary Materials

The following are available online at www.mdpi.com/article/10.3390/genes12091363/s1, Table S1: Differentially expressed genes (DEGs) detected at FDR < 0.1, Table S2: Gene ontology analysis of overlapping DEGs, Table S3: Position of each gene after sum and product of ranking of downregulated and upregulated genes from the four datasets, Table S4: Gene ontology analysis of top 5% ranking genes, Table S5: Cell type enrichment results (Zeisel et al. 2018, 265 cell types), Table S6: Cell type enrichment results (Saunders et al. 2018, 565 cell types), Table S7: FUMA cell type analysis on GWAS Summary Stats, Table S8: Gene-set enrichment analysis of MIA gene-sets using human GWAS results, Table S9: Full denovolyzeR results (Competitive).
